# On the timing of interventions to preserve hospital capacity: lessons to be learned from the Belgian SARS-CoV-2 pandemic in 2020

**DOI:** 10.1186/s13690-021-00685-2

**Published:** 2021-09-13

**Authors:** Christel Faes, Niel Hens, Marius Gilbert

**Affiliations:** 1 I-BioStat, Data Science Institute, Hasselt University, Hasselt, Belgium; 2 Centre for Health Economic Research and Modelling Infectious Diseases, Vaccine and Infectious Disease Institute, University of Antwerp, Antwerp, Belgium; 3 Spatial Epidemiology Laboratory, Université Libre de Bruxelles, Brussels, Belgium; 4 Fonds National de la Recherche Scientifiques, Brussels, Belgium

**Keywords:** SARS-CoV-2, Hospital load, Interventions, Phase diagram

## Abstract

Using publicly available data on the number of new hospitalisations we use a newly developed statistical model to produce a phase portrait to monitor the epidemic allowing for assessing whether or not intervention measures are needed to keep hospital capacity under control. The phase portrait is called a *cliquets’ diagram*, referring to the discrete alarm phases it points to. Using this cliquets’ diagram we show that intervention measures were associated with an effective mitigation of a Summer resurgence but that too little too late was done to prevent a large autumn wave in Belgium.

**Supplementary Information:**

The online version contains supplementary material available at 10.1186/s13690-021-00685-2.

## Background

Despite different testing strategies as well as controversy with respect to (over) counting deaths, Belgium has been hit particularly hard by the coronavirus, placing the country near the top in international rankings when looking at the official number of confirmed cases per 100,000 and the official number of deaths per million [1]. On December 6, 2020, Belgium accounted for more than half a million confirmed cases and over 17,000 SARS-CoV-2 confirmed and suspected deaths. There are several factors explaining the vulnerability of Belgium to the SARS-CoV-2 pandemic including high international mobility as a result of Belgium’s location at the centre of Europe and with Brussels being the capital of the European Union, as well as a high population density, high average household size and an older population structure that combined with a relatively high mixing behaviour increases transmission potential and the associated disease burden [1, 2].

Belgium has known three surges of the coronavirus in 2020 (see Figure A1 in Additional file 1). The large number of hospitalizations of covid-19 patients has twice forced hospitals to postpone regular care of non-covid-19 patients. The first wave occurred between March 8 and June 1, accounting for a total of 58,641 confirmed cases with testing mostly focusing on severe illness, 17,132 hospitalizations and 9,377 deaths. Based on the national surveillance of covid-19 hospitalizations in Belgium [3, 4] the median age of hospitalized patients was estimated as 70 years (interquartile range (IQR) 55-82). In the summer period, between July 1 and August 31, a local increase in confirmed cases was observed in the province of Antwerp and Brussels (24,056 confirmed cases in Belgium, of which 49.6% occurred in Antwerp and Brussels). It was mainly younger people who got infected in this period (median age 52 years, IQR 33-76), resulting in less severe infections and a smaller number of hospitalizations (1,220 hospitalizations), but it did put high pressure on general practitioners. A second large wave started on October 1, with 455,442 confirmed cases, 22,126 hospitalizations and 6,817 deaths on November 30. While confirmed cases are younger in this time period (median age 43, IQR 27-59), the age of hospitalisation is similar as in the first wave (median age 71, IQR 57-82). Changes in the testing strategy over time make comparisons of the number of confirmed cases difficult, but the number of hospitalizations is a more stable and important indicator of the severity of the outbreak and has a direct impact on the hospital capacity [5].

## Methodology

Using publicly available data from Sciensano, the Belgian institute for health, on the number of new hospitalisations we develop a statistical model from which a phase portrait is defined to monitor the epidemic, allowing for assessing whether or not intervention measures are needed to keep hospital capacity under control [4]. Note that all Belgian hospitals have to report the number of hospitalized covid-19 patients to Sciensano through a daily online survey [6]. The phase portrait is a representation of the trajectory of the epidemic with respect to the number of covid-19 hospitalizations. The diagram uses the average of the daily number of new hospitalizations on a 7-day sliding window and the growth rate based on the past 14-days new hospitalizations. The growth rate is estimated based on a normal regression model of the log10-transformed number of hospitalizations (see Additional file 2 for details). For each combination of new hospitalizations and growth, the expected ICU load is projected for a 14-days horizon, from which the number of patients requiring intensive care is predicted based on the distribution of time spent in the intensive care unit (ICU) [3]). The projection of ICU load is obtained based on a convolution of the new patients on time *t-i* and the probability that the patients spends *i* days in ICU.

The hospital contingency plan was proposed in Belgium by the Hospital and Transport Surge Capacity Committee [7]. This is an advisory body that recommends taking adequate control measures for hospitals and patient transport capacity. The plan consists of 5 different phases while focusing on covid-19 related ICU care: In Phase 0, 15% of ICU beds are reserved for covid-19 patients (303 ICU-beds); in Phases 1A and 1B, this is increased to, respectively, 25% (528 ICU-beds) and 50% (987 ICU-beds) of the normal ICU bed capacity; in Phases 2A and 2B, 60% of the normal ICU capacity is reserved for COVID patients, and in addition there is an upscaling of the number of ICU beds, by creating a supplementary 15% ICU beds for covid-19 patients in Phase 2A (1502 ICU-beds), and 40% in Phase 2B (2019 ICU-beds). Note that within this scheme the total number of patients (covid-19 and non-covid-19) in ICU moves from 2001 (Phase 0, 1A & 1B) to 2304 (Phase 2A) and 2821 (Phase 2B) and consequently yields a gradual decrease in non-covid-19 ICU capacity.

The cliquets’ diagram shows - from green to red - the severity of the outbreak in terms of hospital and future covid-related ICU load. The green region can be considered a “safe zone” in which the number of new hospitalisations is limited with a decrease (growth <1) or a limited increase (growth >1). This zone is associated with a limited number of covid-19 patients at ICU (<50 ICU beds in the next 14 days, a somewhat ad-hoc choice for the first part of phase 0). Next is the yellow region, a region of increased vigilance (second part of phase 0). The orange (phase 1A & 1B) and red (phase 2A & 2B) regions are “high impact” and “no-go” zones, in which non-covid-19 care decreases substantially and additional capacity for covid-19 needs to be provided for.

Validation of the method has been investigated and the main results are included in Additional file 3.

## Results

**Fig. 1:**
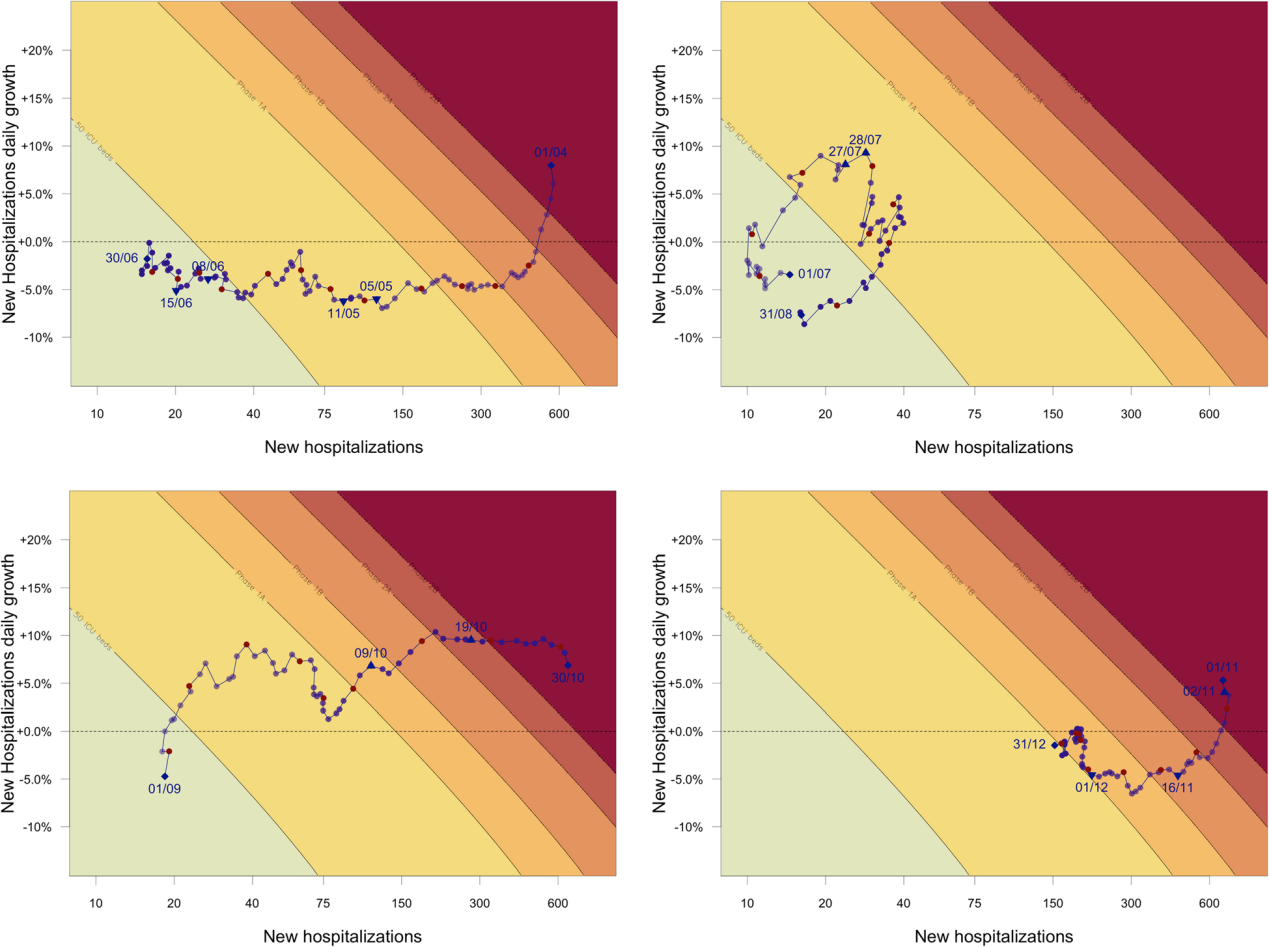
Cliquets’ diagram for different time periods: **A** left upper panel: April 1 to June 30, **B** right upper panel: July 1 to August 31, **C** lower left panel: September 1 to October 31, **D** lower right panel: November 1 to December 31. Red points indicate the midpoint of the weeks (Wednesdays), triangles correspond to the given dates of increased measures (triangle pointing upwards) or released measures (triangle pointing downwards). Dates are given in the format day/month

A key question is whether the resurgence in hospitalisations in October 2020 could have been foreseen. Therefore we look at the phase portraits over four consecutive time periods throughout the pandemic (Fig. 1). The top left figure (Fig. 1A) shows the situation from April 1 until June 30, which is the period from the peak of the first wave until the end of the first wave. The exit strategy to release restriction measures was carefully designed in this period (for a general overview of the main principles see [8, 9], page 35) and consisted of 4 exit-phases (exit-phase 1a: May 5 with start of contact tracing, exit-phase 1b: May 11 with reopening of schools and gradual reopening of schools, exit-phase 2: June 8 with reopening of bars and restaurants, exit-phase 3: June 15 with reopening of borders within the EU, exit-phase 4: July 1 with increasing the limit on number of close contacts). The cliquets’ diagram shows that the growth of hospitalizations was declining as of April 1 and that the number of hospitalizations steadily decreased, such that we moved from the no-go-zone for ICU capacity towards the safe zone, which was reached early in June. The exit strategy was largely successful in reducing the number of hospitalisations and keeping those numbers under control.

The top right figure (Fig. 1B) shows the summer period, from July 1 until August 31. The number of new hospitalisations and growth in hospitals was kept under control until July 23. Thereafter the increase in the number of hospitalisations followed an asynchronous increase of the number of confirmed cases in the Provinces of Antwerp (starting July 12) and Brussels (starting July 27) which was successfully curbed in August following the instalment of additional control policies on July 27 at the national level (stricter rules on wearing mouth masks) and more strict measures in the province of Antwerp on July 28 (reduction of number of contacts and curfew), which is translated into a loop in the phase portrait (Fig. 1B) [10]. So, early and strong interventions when the trajectory entered the yellow region were successful in curbing the epidemic and bringing it back under control. Note that such a wave is described by a clockwise circular movement on the cliquets’ diagram.

The bottom left figure (Fig. 1C) shows the time period from September 1 until October 31. In the course of September, an increase in the number of new hospitalisations was observed again following an increase in the number of confirmed cases at the end of August. However, this time, no new policy measures were taken until October 9 (mainly reduction of number of close contacts) when the trajectory had entered the orange zone, and the measures did not result in a decrease in the number of new hospitalisations after which the autumn wave followed ([10], page 29). A considerable decrease in the daily growth of the number of new hospitalisations was observed late September, possibly due to the combination of a large amount of incoming international travel at the end of the summer holidays combined with the restart of schools and universities in September, but also linked to adhesion and motivation.

The bottom right figure (Fig. 1D) presents the period from November 1 until December 31. The situation in Belgium did worsen quickly and hospital networks moved from Phase 0 to Phase 1A (yellow to orange in the diagram), from Phase 1A to Phase 1B (dark orange) and eventually to Phase 2A (red). Intervention measures were implemented on October 19 (closing of bars and restaurants) and additional measures on November 2 (partial lock-down with e.g. closure of shops, schools and further reduction of number of contacts allowed), resulting in a slowing down of the growth of new hospitalizations followed by a decrease in new hospitalizations [10]. On November 16, primary schools and first grade secondary schools were fully reopened while second and third grade schools were partially reopened, and on December 1 shops were reopened [10]. About two weeks later, the growth in hospitalizations increased again, resulting in an upward movement in the diagram from the beginning of December.

## Discussion

We evaluated the status of the SARS-CoV-2 pandemic in Belgium using a simple phase portrait depicting the number of new hospitalisations versus the daily growth rate of new hospitalisations and predicting the number of covid-19 patients requiring intensive care. We focused on the time period in which variants had little impact, and the flow from hospital to intensive care stayed approximately constant.

Dividing the pandemic in different time periods and using the cliquets’ diagram shows a clear association between the intervention measures in August, i.e. in the yellow area, and gaining control over the pandemic. The decrease in the number of hospitalizations is possibly strengthened by other behavioral factors, but both direct and indirect impact of the intervention measures are expected. In September-October, however, there was a substantial increase in the number of new covid-19 hospitalisations whereas new non-pharmaceutical interventions only started when entering in the orange “high impact” area. Moreover, these interventions appeared to be insufficiently strong to curb the epidemic. It is important to note that the inaction in September-October coincided with a transition from a temporary federal government to the installment of a definitive one and a high level of scepticism toward the reality of the resurgence of the epidemic by several experts in the social and conventional media.

The cliquets’ diagram is merely a visualisation of the epidemiological situation in hospitals. But it allows for simultaneously visualizing where we are in terms of speed (new hospitalizations per day) and acceleration (daily growth rate) of the epidemic with a forward thinking toward the 14-days horizons covid-19 ICU occupancy. The historical situation shows that interventions taken early in the yellow region were associated with keeping the hospital capacity under control. Note that Belgium, relative to other countries, has a relatively large ICU capacity which could have led to overconfidence in policy control whereas early intervention is key given that with low numbers mitigation strategies are much more effective.

There are several limitations related to the proposed cliquets’ diagram. First, we relied on the daily number of new covid-19 hospitalisations which are available for Belgium through a daily hospital surge survey developed and implemented by the national public health organization Sciensano and for which hospitals provided timely input [4]. This may not be available for other countries. Second, using new hospitalisations yields a more stable, but somewhat late indicator. Combining the growth rate based on, e.g., confirmed cases, gives a lead time, which we estimated to be 7-10 days, (results not shown). We believe delays and underreporting in the number of confirmed cases doesn’t have a large impact given that changing case definitions and test saturation are only likely to occur when already in a high-impact or no-go zone. Using test positivity rates could provide a useful addition to the number of confirmed cases. Further research includes defining a phase portrait based on confirmed cases though the connection to the hospital contingency phases is less straightforward because of the age-specificity of hospitalisation rates. Finally, it is important to mention that whereas we relate the epidemiological situation to intervention measures; we cannot assume causality and thus careful interpretation is warranted.

## Supplementary Information

**Additional file 1.**

**Additional file 2.**

**Additional file 3.**

## Data Availability

Hospital data are made publicly available by Sciensano at https://epistat.wiv-isp.be/covid/. The code is available at https://github.com/NielHens/Cliquets.

## Abbreviations

IQR: Interquartile range
ICU: Intensive care unit
