# Supplementary material for: On the timing of interventions to preserve hospital capacity: lessons to be learned from the Belgian SARS-CoV-2 pandemic in 2020
Source: Arch Public Health. 2021 Sep 13;79:164. doi: 10.1186/s13690-021-00685-2 (PMC8436011; doi:10.1186/s13690-021-00685-2)
Supplement: Supplementary file 1 — Additional file 1. [file 13690_2021_685_MOESM1_ESM.docx]

**Additional File 1**

**
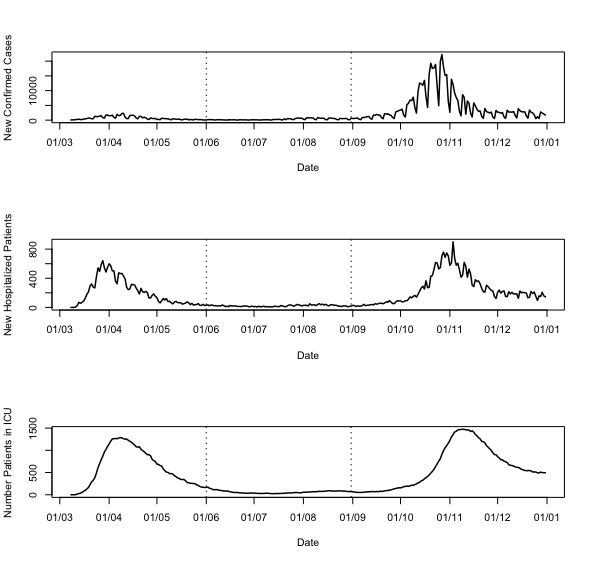
**

**Figure A1:** covid-19 epidemic curve in Belgium in 2020. Top: daily number of new confirmed cases, Middle: daily number of new hospitalized patients due to covid-19, Bottom: daily total number of patients in ICU due to covid-19. The three surges of the coronavirus in 2020 are highlighted using the vertical lines.
