# Supplementary material for: On the timing of interventions to preserve hospital capacity: lessons to be learned from the Belgian SARS-CoV-2 pandemic in 2020
Source: Arch Public Health. 2021 Sep 13;79:164. doi: 10.1186/s13690-021-00685-2 (PMC8436011; doi:10.1186/s13690-021-00685-2)
Supplement: Supplementary file 2 — Additional file 2. [file 13690_2021_685_MOESM2_ESM.docx]

**Additional File 2**

Publicly available data from Sciensano are used on the number of new hospitalisations, total number of hospital beds and total number of intensive care (ICU) beds, which are reported on a daily basis (www.sciensano.be).

Data are presented as the pair of new hospitalizations and growth rate of new hospitalizations. The new hospitalizations are averages of the past 14-day number of new hospitalizations. The growth rate is obtained from modeling the past 14-day number of new hospitalizations $H\left( t \right)$ as

$$log_{10}(H\left( t \right)) \sim N(\alpha+\beta t+\gamma w_{t},\sigma^{2})$$

with $w_{t}$ an indicator for weekends and holidays. The growth rate is then calculated as

$$g={10}^{\beta}$$

Colours of the phase diagram are based on the predicted ICU capacity. For an assumed number of new hospitalizations $H\left( t_{0} \right)$ on day $t_{0}$ and and assumed (constant) growth rate $g$, we calculate the number of new hospitalizations in the next $t$ days as

$$H\left( t \right)=H\left( t_{0} \right)g^{t-t_{0}}$$

Based on the predicted number of new hospitalisations, we derive the total number of patients in intensive care unit (ICU), based on the proportion of hospitalised patients that need intensive care and the length of stay in ICU. The number of patients in ICU on day $t$ is

$$ICU\left( t \right)=\pi\sum_{i} H\left( t-i \right)\delta\left( i \right)$$

where $\pi$ is the fraction of hospitalized patients going to ICU and $\delta\left( i \right)$ is the probability that a patient stays $i$ days in ICU.

The distribution of length of stay in ICU is derived from a multicenter registry in Belgium that collects information on hospital admission related to covid-19 infection, for the period March to August 2020 [5,6]. Patients staying longer than 60 days in ICU are assumed to leave ICU on day 60. The fraction $\pi$of hospitalized patients that need intensive care is, based on the survey, 0.152. As this is a survey, and does not contain all hospitalizations, this is corrected by the fraction of daily ICU beds reported in the hospital survey versus the daily reported national ICU load (1/2.15) and the fraction of hospital beds reported in the survey and reported nationally (1/1.33). As a result, we estimate $\pi$ as 0.245.

As cutpoints, we use the estimated ICU load on day $t_{0}+14$ (which resembles the covid-19 ICU load if the behavior stays the same in 2 weeks). Cutpoints are chosen in correspondence to the hospital contingency plan in Belgium consisting of 5 different phases while focusing on covid-19 related ICU care: Phase 0: 303 ICU-beds; Phase 1A: 528 ICU-beds; Phase 1B: 987 ICU-beds; Phase 2A: 1502 ICU-beds; Phase 2B: 2019 ICU-beds. Note that within this scheme the total number of patients (covid-19 and non-covid-19) in ICU moves from 2001 (Phase 0, 1A & 1B) to 2304 (Phase 2A) and 2821 (Phase 2B) and thus non-covid-19 care gradually decreases with an increase in phases.

The code is available at <https://github.com/NielHens/Cliquets>.
