# Supplementary material for: On the timing of interventions to preserve hospital capacity: lessons to be learned from the Belgian SARS-CoV-2 pandemic in 2020
Source: Arch Public Health. 2021 Sep 13;79:164. doi: 10.1186/s13690-021-00685-2 (PMC8436011; doi:10.1186/s13690-021-00685-2)
Supplement: Supplementary file 3 — Additional file 3. [file 13690_2021_685_MOESM3_ESM.docx]

**Additional File 3**

For each combination of the number of new hospitalizations and growth in hospitalization, a projection of the ICU load is made for 2 weeks later. The colours of the cliquets’ diagram correspond to this projection. To validate the proposed method, we make a scatter plot of the predicted versus observed ICU load, which is presented in Figure C1. In addition, we add, in the diagram, the actual ICU capacity that was observed 14 days later. For clarity, the ICU capacity is only included on Wednesdays. Figures C2-C5 correspond to the 4 time periods that were used in Figure 1.

Based on Figure C1, we see good agreement between the observed and predicted ICU load. The corresponding correlation is 82%. There is an overestimation when the ICU capacity is high. This is not unexpected, as the prediction is made under the assumption that the growth remains constant for the next 2 weeks, which is very unlikely as strict measures were taken when ICU capacity was high.


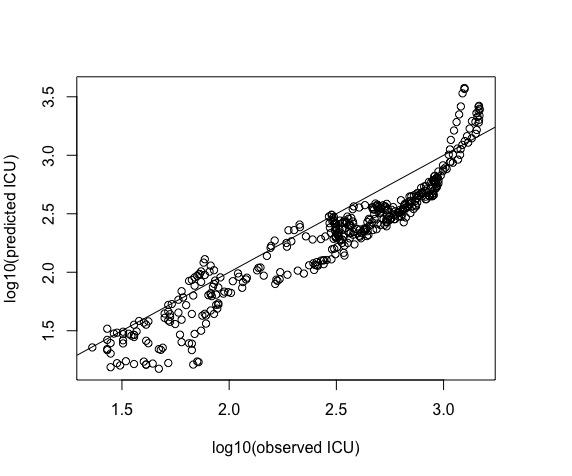


Figure C1: Projection of ICU load for the period April 1, 2020 until June 4, 2021

Figures C2-C5 show that the classification of the time points according to the projected ICU capacity works well. The rate of correct classification is 66%. Of course, it is not a perfect classification, as the impact of policy measures and individual behaviour plays an important role, and can cause deviations in the growth rate.


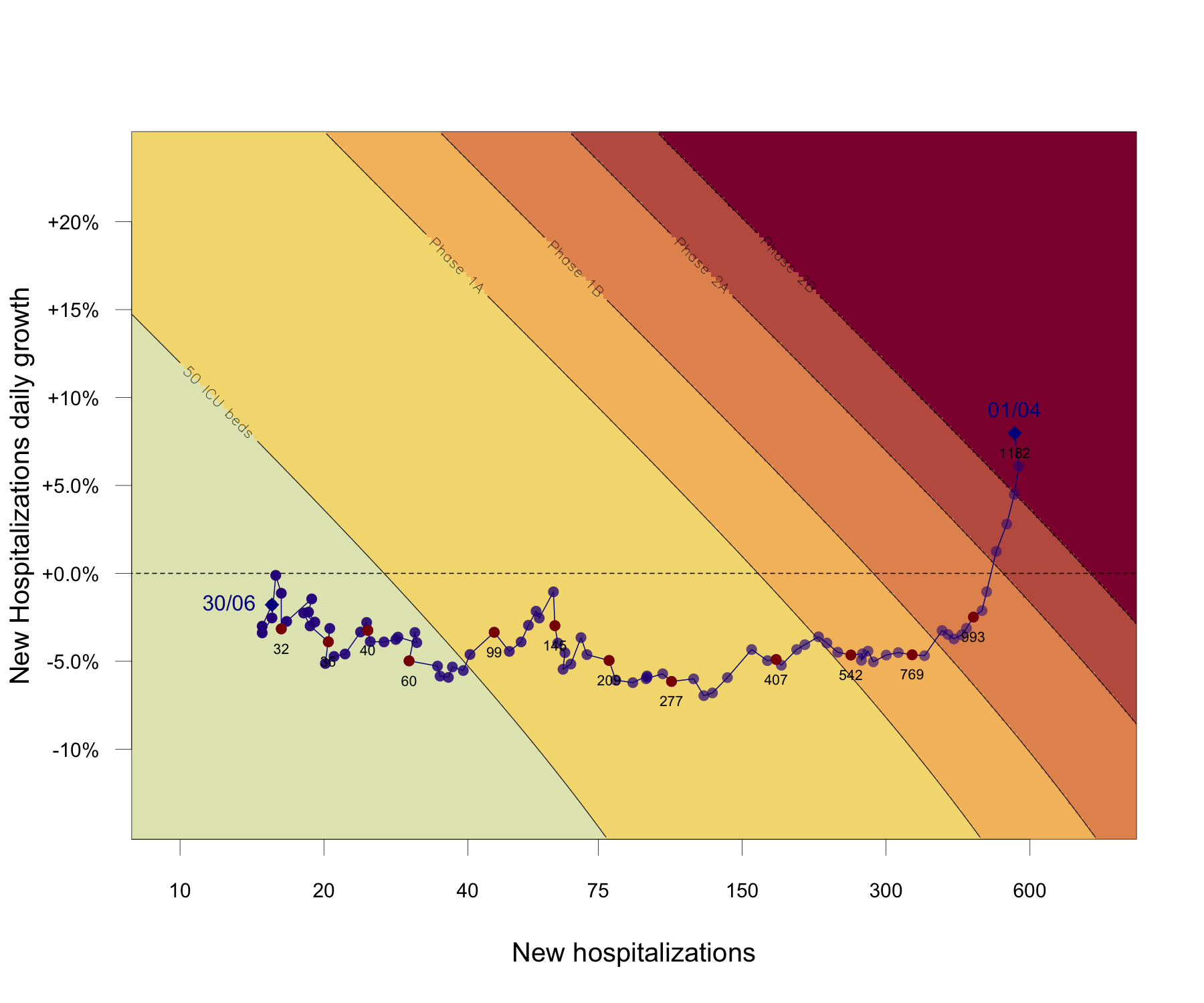


Figure C2: Projection of ICU load for the period April 1-June 30


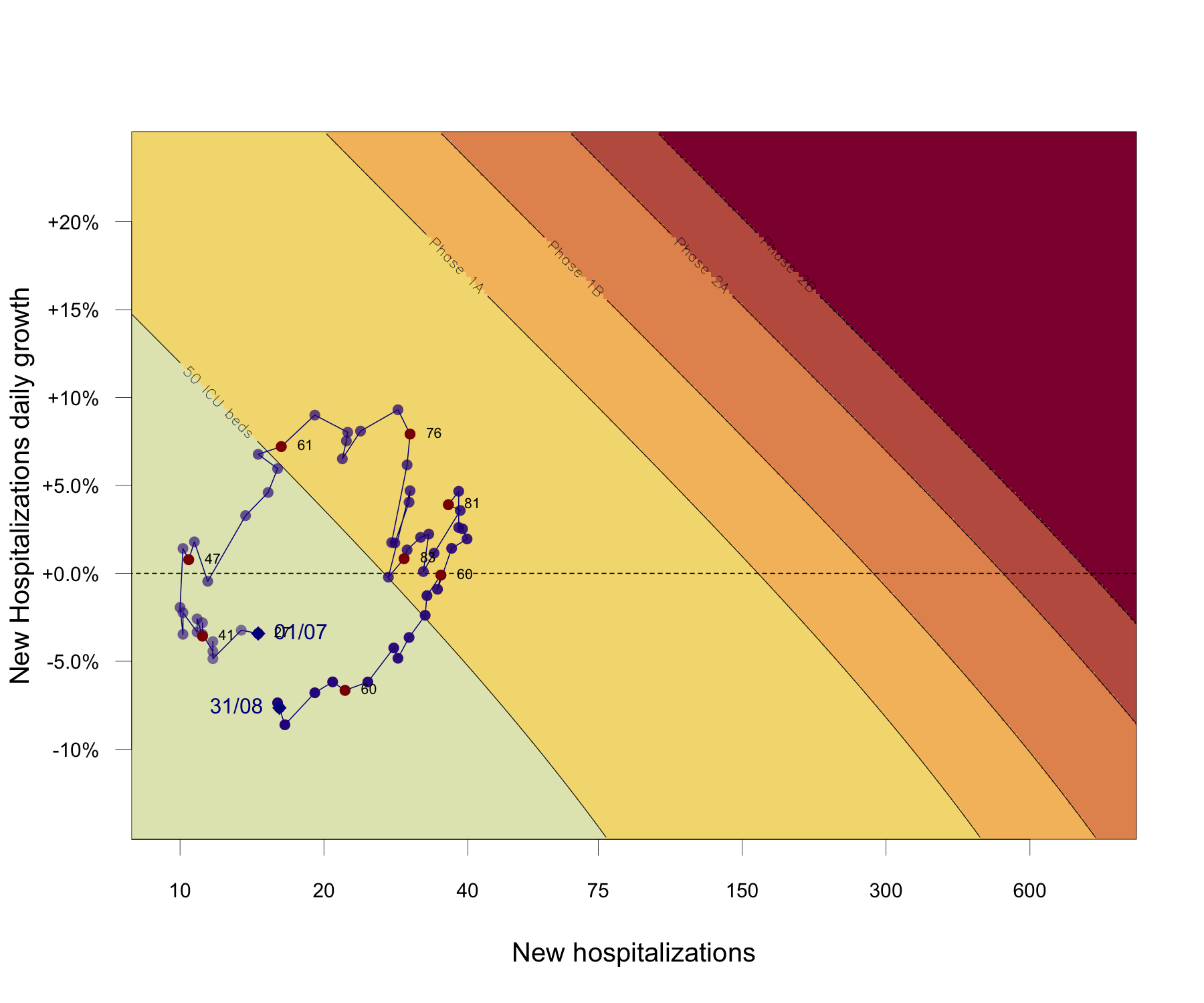


Figure C3: Projection of ICU load for the period July 1-August 31


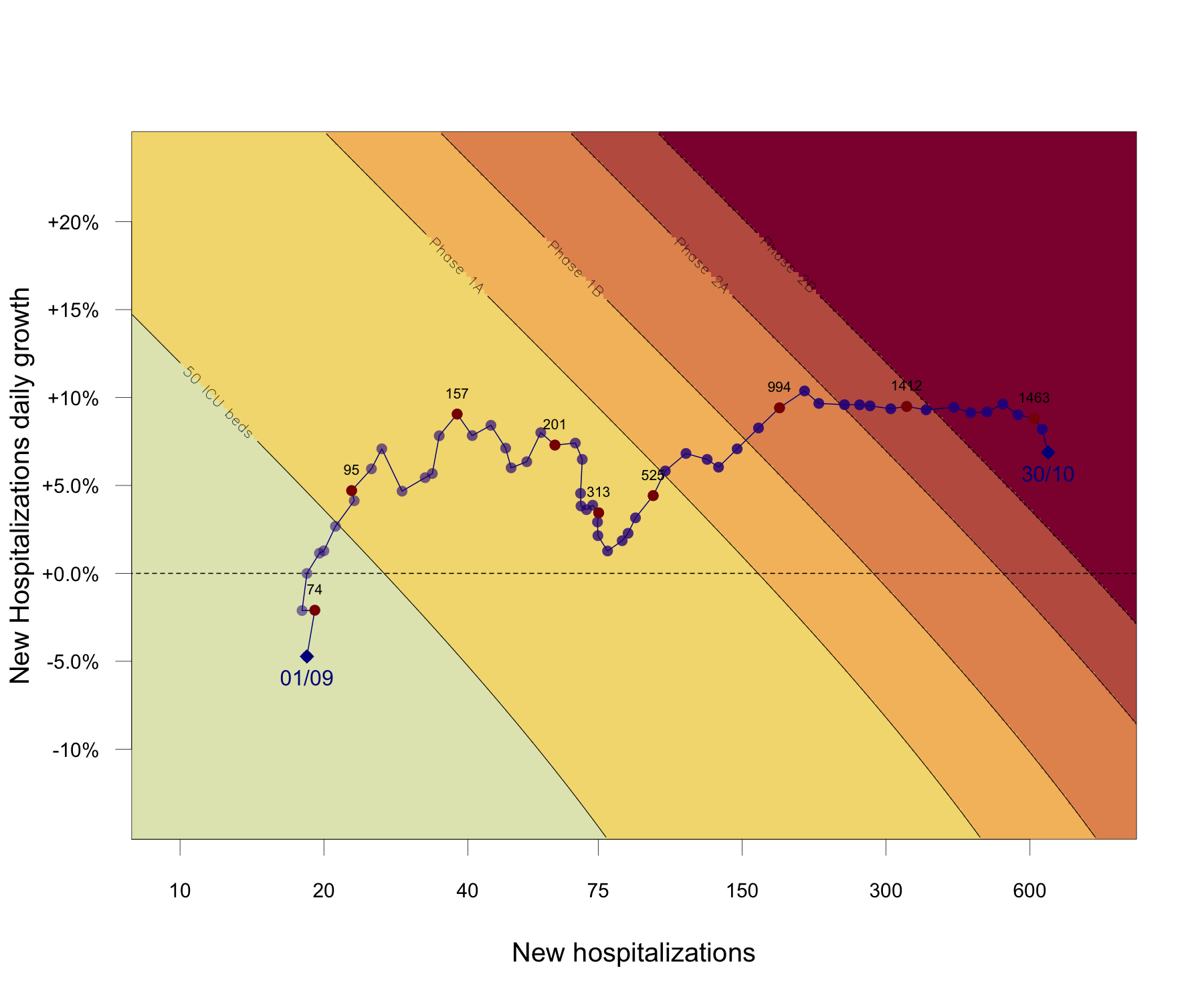


Figure C4: Projection of ICU load for the period September 1-October 30


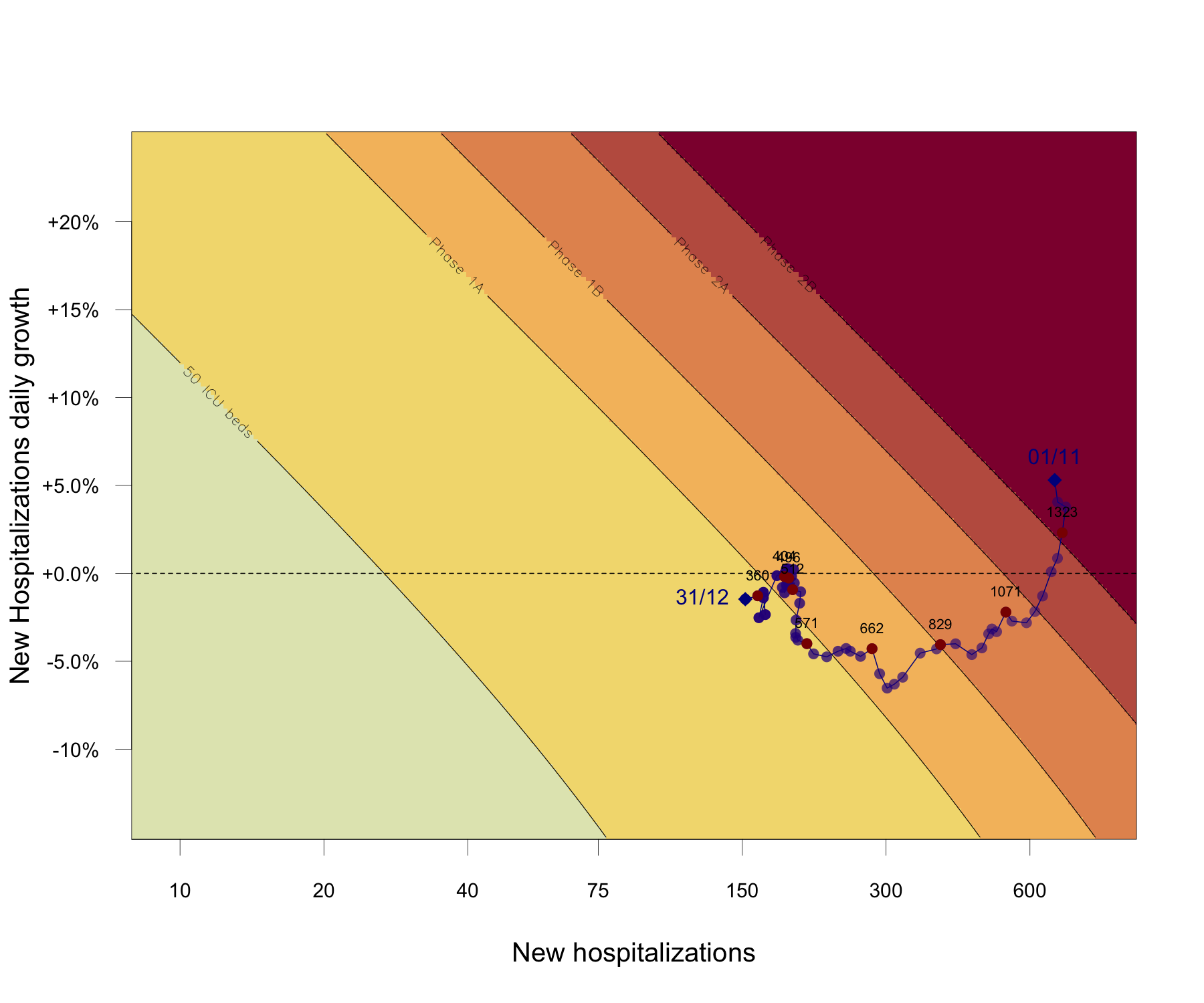


Figure C5: Projection of ICU load for the period November 1-December 12
